# Fabrication of Polymer/Cholesteric Liquid Crystal Films and Fibers Using the Nonsolvent and Phase Separation Method

**DOI:** 10.1021/acs.langmuir.4c01759

**Published:** 2024-06-25

**Authors:** Tzu-Hsun Kao, Hsun-Hao Hsu, Jui-Juin Chen, Lin-Ruei Lee, Hui-Yu Chen, Jiun-Tai Chen

**Affiliations:** † Department of Applied Chemistry, National Yang Ming Chiao Tung University, Hsinchu 300093, Taiwan; ‡ Department of Physics, National Chung Hsing University, Taichung City 402204, Taiwan; § Center for Emergent Functional Matter Science, National Yang Ming Chiao Tung University, Hsinchu 300093, Taiwan

## Abstract

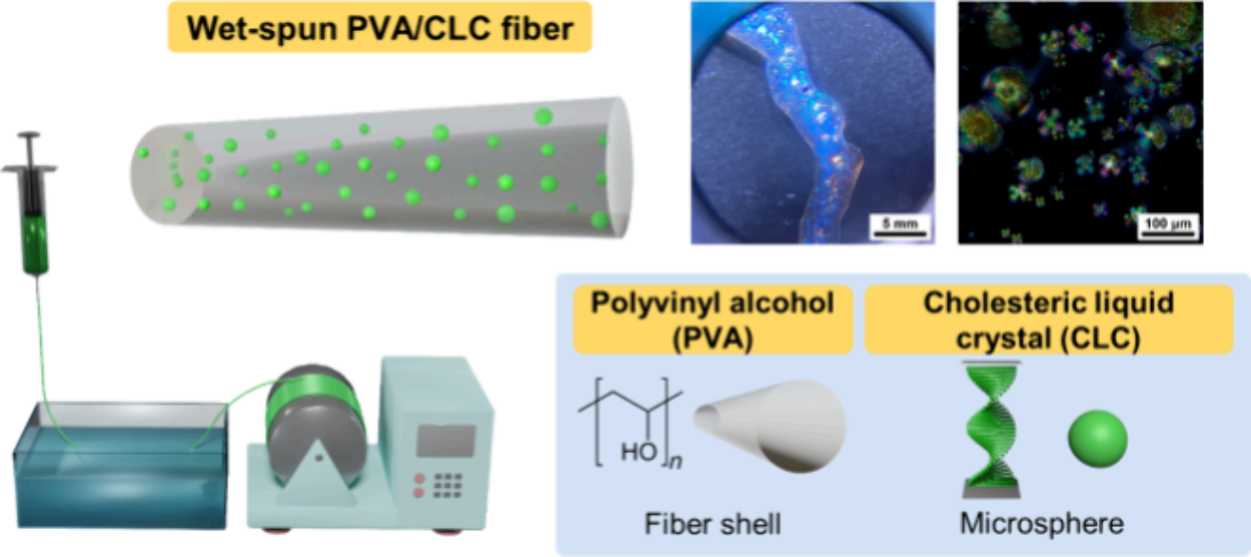

In recent years, liquid crystal materials have drawn great interest because of their wide range of applications. Among various thermochromic materials, cholesteric liquid crystalline (CLC) materials have been well studied and reported. CLC materials have the advantages of ready manipulation and multiple color transitions. For the further development of smart clothing and wearable electronics, however, the incorporation of CLC materials into polymers still remains challenging. The difficulties lie in the prevention of leakage of CLC and retention of the cholesteric liquid crystalline phase. In this work, we demonstrate a versatile nonsolvent and phase separation method using polar solvents to incorporate CLC microspheres into polymer matrix. Poly(vinyl alcohol) (PVA), a water-soluble polymer, is chosen as the polymer because of its high transparency and ease to handle. Using spin-coating and wet spinning techniques, PVA/CLC films and fibers can be fabricated. The formation of CLC microspheres in the polymer matrix is characterized through optical and polarized microscopy. Compared with the CLC films, the PVA/CLC composites demonstrate superior thermal stability. Moreover, both PVA/CLC films and fibers exhibit good color stability from the electrical tests. This work provides an effective strategy to prepare polymer/CLC composites, paving a wide avenue toward applications in smart textiles, display technologies, and medical devices.

## Introduction

Liquid crystal materials have been extensively studied because of their unique optical properties and applications,^1^ such as liquid crystal displays,^2^ smart glasses,^3^ and e-paper.^4^ Furthermore, unique features of liquid crystals have been harnessed, such as thermochromism,^5^ electrochromism,^6^ and pressure-induced color change.^7^ In recent years, increasing research has focused more on combining liquid crystal materials and polymers, including liquid crystal elastomers,^8^ polymer-dispersed liquid crystals,^9,10^ and, most importantly, liquid crystal fibers.^11^ While the diverse applications are crucial for modern technology, challenges remain in integrating liquid crystals into polymer matrices. The issues include the light leakage of liquid crystals and destruction of the liquid crystal phase.^12^

Previous studies have presented various approaches for creating polymer/liquid crystal fibers, especially using cholesteric liquid crystals (CLC) because of their advantages of ready manipulation and multiple color transitions. For example, Lagerwall et al. used a solvent to mix CLCs with polyvinylpyrrolidone (PVP), fabricating fibers through coaxial electrospinning.^13^ This method inherently used high electric fields, forcing the solution to accumulate charges and to eject from the needle tip, which then formed PVP/liquid crystal fibers with a diameter of approximately 7 μm. This technique, however, requires careful control of conditions for electrospinning. Also, inconsistencies in the liquid crystal distribution were commonly observed in the electrospun fibers. Moreover, these fibers suffered from low mechanical strength and inconsistent coaxial conditions, making the fibers prone to the leakage of liquid crystals. Another coaxial electrospinning technique was adopted by Tang et al. to create thermochromic CLC fibers.^14^ They employed a miscibility technique, blending CLCs with polystyrene using solvents for the electrospinning process. These fibers had an average diameter of about 23 μm, but the orderly structures of CLC were disrupted by dissolution of the solvent, impairing their color display capabilities. To prevent the leakage of liquid crystals, Fu et al. demonstrated intelligent functional fibers embedding CLC microcapsules.^15^ The emulsion polymerization technique was utilized to encapsulate the CLCs. After the microcapsule was blended with PVP, the electrospinning method was implemented to create fibers around 10 μm in diameter. While this method effectively prevented liquid crystal leakage, the fabrication process was complex, and the upscaling of the method remained uncertain.

To address the above issues, in this work, we present a simple and efficient nonsolvent and phase separation method using polar solvents to produce polymer/CLC composite films and fibers. A CLC is prepared by mixing a left-handed chiral dopant, known as 4′-(1,3-dimethyl-3-chloro)propoxy-4-cyanobiphenyl (NYCL), with a commercial nematic liquid crystal DLC-111, forming a blue phase at a higher temperature. A water-soluble polymer, poly(vinyl alcohol) (PVA), is selected as the polymer matrix because of its high transparency and easiness to handle. By leveraging the hydrophobic properties of CLC using polar solvents, CLC microspheres can be stabilized in the aqueous PVA solution. Through spin-coating and wet spinning techniques, PVA/CLC films and fibers can be fabricated, in which the CLC microspheres are embedded in the PVA matrix. The wet spinning method provides a wider operation window to prepare the fibers compared with that of the electrospinning method. Optical and polarized microscopy and UV–vis spectroscopy are used to characterize the optical properties of the CLC microspheres in the PVA matrix. Because of the protection of the PVA matrix, no destruction of the CLC structure is observed. Compared with CLC films, the PVA/CLC composites exhibit better thermal stability. Besides, from the electrical tests, both PVA/CLC films and fibers show good color stability. Overall, this work introduces an efficient nonsolvent and phase separation method for producing PVA/CLC films and fibers, opening doors for applications in smart textiles, display technologies, and medical devices.

## Experimental Section

### Preparation of the CLC Cells

Commercial nematic liquid crystal DLC-111 was blended with a left-handed chiral dopant, known as 4′-(1,3-dimethyl-3-chloro)propoxy-4-cyanobiphenyl (NYCL), in a test tube in a weight ratio of 15:85. DLC-111 was obtained from Daxin Materials, and NYCL was sourced from Daily Polymer.^16^ This mixture was then transferred to a thermal shaker (TS-100, Biosan) set at a temperature of 80 °C and a shaking rate of 800 rpm for 3 h to achieve a homogeneous state, forming the CLC material. A cell was assembled by overlapping two transparent conductive indium tin oxide (ITO)-coated glass substrates with conductive surfaces facing inward without additions of any alignment layers, and the NYCL molecules were used to induce the helix of CLC. The overlapped area was 1.5 × 1.5 cm^2^. The spacing between the substrates was maintained at 30 μm by using polyimide tapes (3M). The uniformity of the spacing was confirmed by the formation of the concentric and uniform Newton’s ring under a coherent light illumination at a wavelength of 530 nm. The cell was then glued on 2 parallel edges using epoxy adhesives and cured for 3 h. After being cooled to room temperature, the CLC mixture was infused into the cell, until the spacing was fully covered. The openings were then sealed with epoxy glue with 3 h of curing. A voltage of 400 V was applied by using a power supply (Extech Electronics Co., EAL-5005). The UV–vis reflectance spectra were measured by using a Hitachi U-3010 spectrometer with an integrating sphere. The optical microscope (OM) (ZEISS, Axiophot) in reflection and polarized-transmission modes was used for morphology observation.

### Preparation and Characterization of the PVA/CLC Films

A 10 wt % solution of poly(vinyl alcohol) (PVA, sourced from Acros Organics, *M*_w_ = 88000 g/mol) was prepared using distilled water and dimethyl sulfoxide (DMSO, sourced from Sigma-Aldrich) with a volume ratio of 3:1 as solvents. The solution was stirred at 300 rpm and held at 80 °C for 48 h. After being cooled to room temperature, the CLC material was added to the PVA solution in a weight ratio of 1:1. This mixture was sonicated for 10 min, forming the PVA/CLC blend. Subsequently, the blend was deposited on an 18 × 18 mm^2^ glass substrate and spin-coated with a 2-step program: 1000 rpm for 60 s and then 3000 rpm for 60 s. The resulting films were dried overnight in ambient conditions for further measurements.

### Preparation of the PVA/CLC Cells

The process of preparing the PVA/CLC cells was similar to that of preparing CLC cells. A cell was created by overlapping two glass substrates coated with transparent conductive indium tin oxide (ITO) with the conductive sides facing each other. The overlapped areas were 1.5 × 1.5 cm^2^. Polyimide tapes (3M) were used to maintain a gap of 30 μm between the substrates. This consistent spacing was verified by observing the formation of concentric and uniform Newton’s rings under a 530 nm wavelength coherent light. The cell was then secured along two parallel edges using epoxy adhesive and left to cure for 3 h. Once cooled to room temperature, the PVA/CLC solution was introduced into the cell until the gap was completely filled. After the openings were sealed with epoxy glue and allowed to cure for 3 h, PVA/CLC cells were obtained.

### Preparation of the Wet-Spun PVA/CLC Fibers

For the wet spinning procedure, the PVA/CLC blend was prepared as previously described but with a PVA concentration of 22 wt %. The PVA/CLC blend was loaded into a plastic syringe attached to a needle with a 1.0 mm inner diameter. Using a syringe pump (KD Scientific), the flow rate of the solution was maintained at 10 mL/min. Acetone (Echo Chemical) was used as a coagulation bath. The fiber collection was achieved using a rotating drum with a diameter of 4 cm rotating at 5 rpm. After collection, the fibers were dried overnight under ambient conditions. The diameters of the fibers ranged from 0.5 to 1.0 mm.

## Results and Discussion

**Figure 1:**
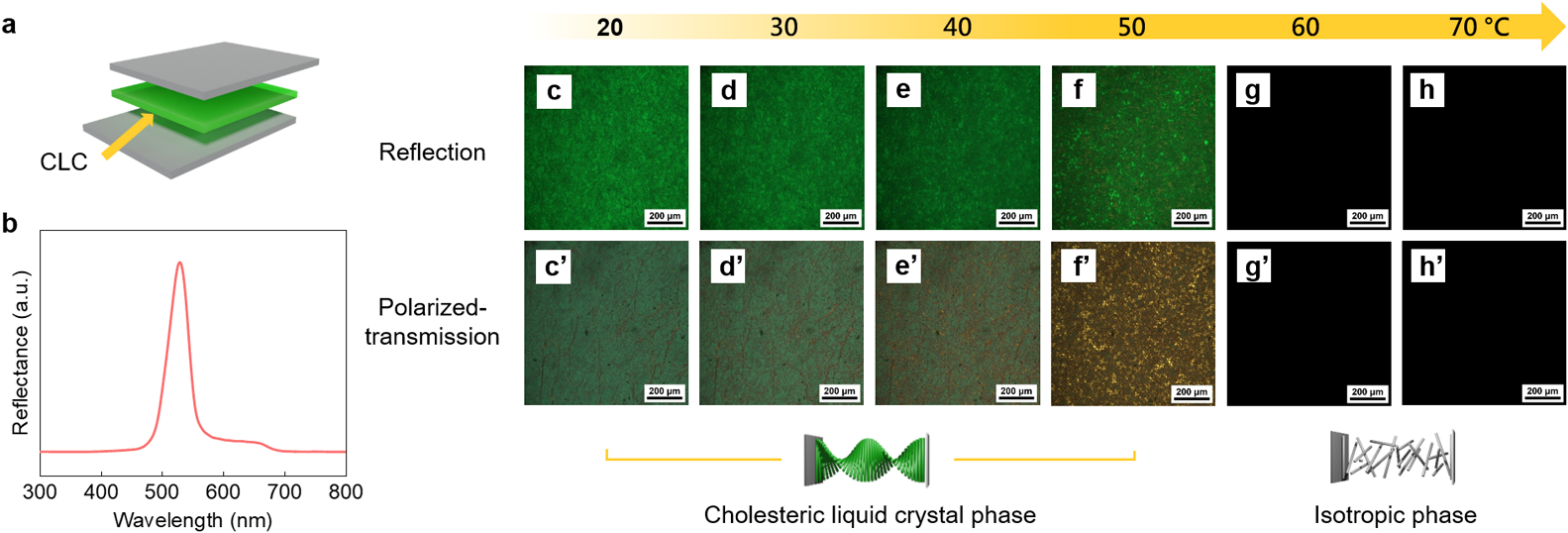
(a) Schematic illustration of a CLC cell. (b) Reflectance spectrum of the CLC. Optical images of the CLC under various temperatures using reflection (c–h) and polarized-transmission modes (c′–h′) from 20 to 70 °C.

Surface-treatment-free CLC cells are made to observe the phases of the CLC (Figure 1a). Because of the high chiral concentrations, the blue phases of the CLC can be induced without applying external electric field.^16^ A left-handed chiral dopant, known as 4′-(1,3-dimethyl-3-chloro)propoxy-4-cyanobiphenyl (NYCL), is mixed with a commercial nematic liquid crystal DLC-111, forming the blue phase. A strong and sharp reflectance at 528.6 nm is observed in the reflectance spectrum (Figure 1b). As shown in Figure 1c–g, when the temperatures fall within the range 20–50 °C, the CLC adopts a cholesteric liquid crystalline state and exhibits a bright green appearance. It is worth noting that the color remains consistent within this temperature range. When the temperature exceeds 50 °C, however, the CLC gains energy with rising temperatures, transforming into an isotropic phase and adopting an irregular molecular arrangement. In this isotropic state, the visible light passes through the CLC without undergoing Bragg’s reflection, making the liquid crystals transparent.

**Figure 2:**
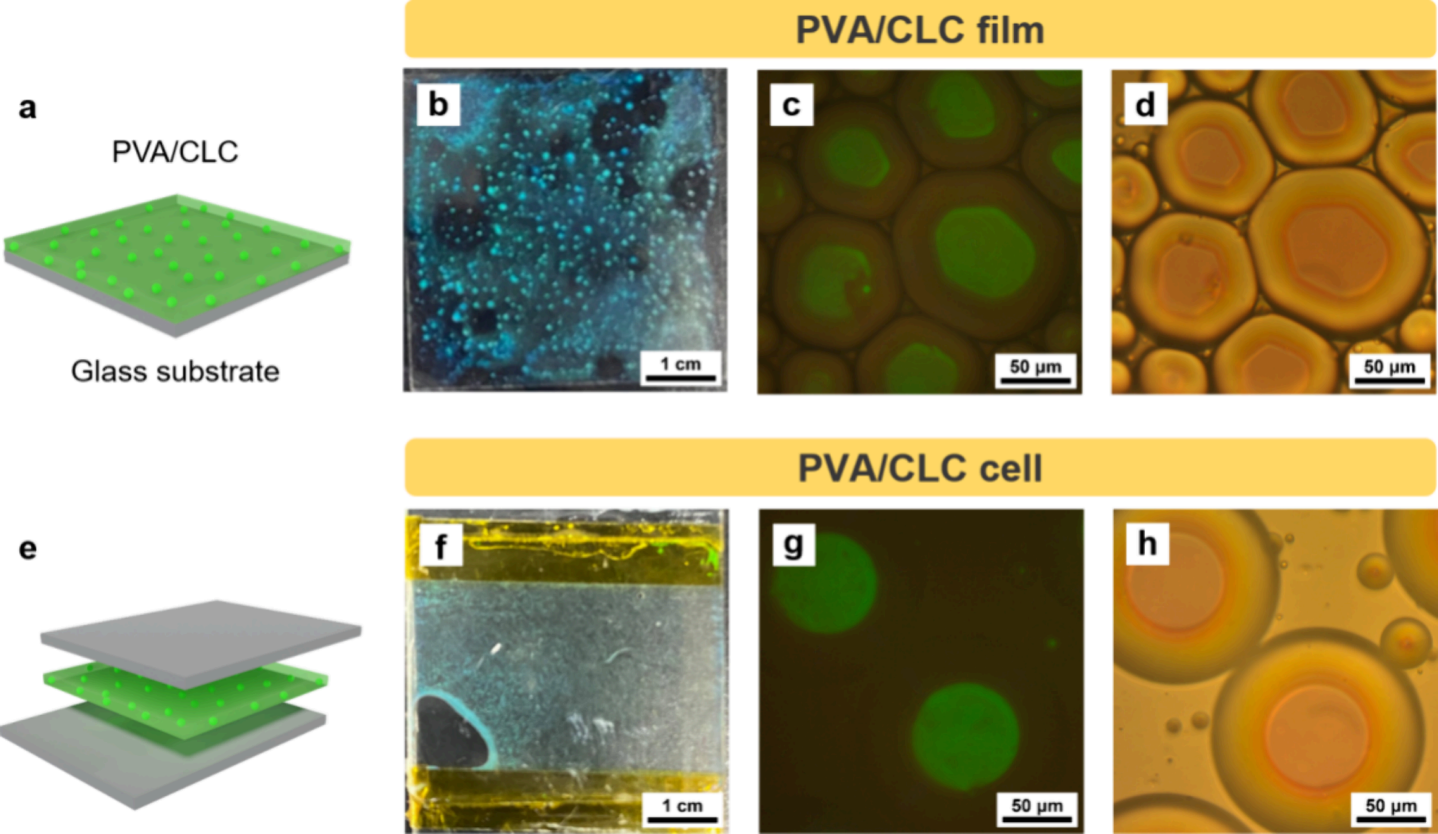
Schematic illustrations of a PVA/CLC film (a) and a PVA/CLC cell (e). Photographs of a PVA/CLC film (b) and a PVA/CLC cell (f). Optical images of a PVA/CLC film and a PVA/CLC cell using reflection (c, g) and transmission modes (d–h).

The goal of creating the PVA/CLC film and cell is to examine possible morphological changes in CLC microspheres under varying environments and to explore the potential future application of the PVA/CLC cell in electronic devices.^17,18^ Additionally, because the PVA/CLC mixture is in a liquid state, we can observe the reflection of light from the CLC microspheres within the liquid state rather than in the solid state (PVA/CLC film). Figures 2a and 2e show the schematic illustration of the PVA/CLC film and PVA/CLC cell. The PVA/CLC films are made by spin-coating the PVA/CLC solution and drying under ambient conditions. Besides, the PVA/CLC cell is obtained by introducing the PVA/CLC solutions into two ITO glasses and sealing the ITO glasses with epoxy glues. To obtain the CLC microspheres, the nonsolvent and phase separation method is selected. Given that the CLC used in our experiments are lipophilic molecules, to prevent any damage to the structure of the CLC from dissolution, water and DMSO are selected as nonsolvents. Although the additions of water and DMSO are necessary, the ratios of H_2_O to DMSO are crucial in the system. Figure S1 shows that the color of NYCL-15% CLC slightly changes with different amounts of DMSO. Furthermore, the color disappears using pure DMSO as solvent, which is caused by the damage of the CLC microspheres. For the corresponding hydrophilic polymer, PVA is selected as our primary polymer to encapsulate the CLC. PVA has a glass transition temperature (*T*_g_) of ∼66.5 °C.^19^ Compared with nonsolvent and PVA, CLC possesses a lower surface energy. Therefore, when nonsolvent and PVA are mixed with CLC, assisted with sonication, the CLC domain reduces the energy by lowering their surface areas, thereby resulting in CLC microsphere structures, accompanied by PVA encapsulation. As shown in Figure 2b,f, microspheres of CLC are observed in the spin-coated PVA/CLC film and cell. For the PVA/CLC films, the average film thickness is found to be between 150 and 200 μm. In Figure 2c, the reflective optical images reveal that the CLCs are distributed in the PVA film in a microsphere form. Additionally, as observed in Figure 2d, CLC microspheres are encapsulated in the PVA layer. The diameters of these microspheres, calculated from the optical microscopy images, range between 20 and 100 μm. The results can be attributed to the combination of the polar cosolvents, PVA, and lipophilic CLC, which leads to phase separation and formation of the encapsulated CLC. Plus, the selection of the polar solvent retains the cholesteric liquid crystalline phase and prevents the formation of the isotropic phase of CLC. As demonstrated in Figures 2g and 2h, even when using liquid crystal cells where the solvents are still present, the CLC continues to form microspheres within the PVA. Additionally, it should be noted that the sizes of microspheres (20–100 μm) in the PVA/CLC film are smaller than those in the PVA/CLC cell (20–150 μm), which might be attributed to the separations and larger distances between the microspheres caused by the confinement of the cell. In the future, we will focus on controlling the sizes of microspheres by incorporating emulsifiers or stabilizers and adjusting the spinning rates during the spin-coating process.

**Figure 3:**
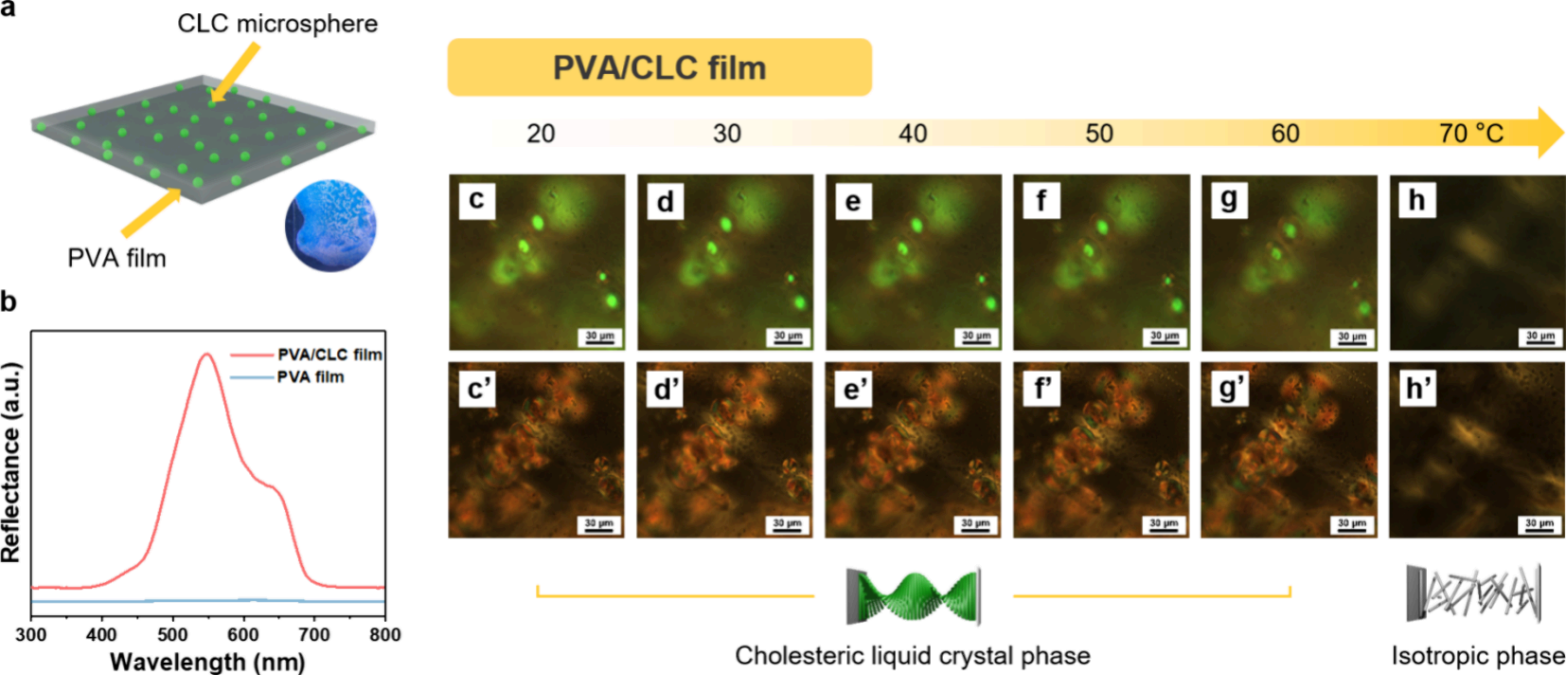
(a) Schematic illustration of a PVA/CLC film. (b) Reflection spectra of PVA/CLC and PVA films. Transmission (c–h) and polarized optical images (c′–h′) of a PVA/CLC film from 20 to 70 °C.

The optical properties of the PVA/CLC and PVA films are analyzed. As shown in the reflection spectrum in Figure 3b, for the PVA/CLC film, a peak wavelength of 543.46 nm indicates the formation of the cholesteric liquid crystalline state. Compared with the reflection peak at 528.26 nm of the CLC film, the increase of the reflection wavelength of the PVA/CLC film could be attributed to the looser packing of the CLC structure. Besides, there is also a shoulder peak observed at 648.50 nm, indicating that there might be another kind of looser packing between the CLC molecules stabilized by the PVA film. In contrast, the pure PVA film exhibits no reflectance in the visible light spectrum at room temperature, suggesting that the PVA does not hinder the reflection of light from the CLC microspheres. To investigate the effect of the PVA encapsulation on the CLC microspheres, the PVA/CLC films are heated to 70 °C and then cooled to room temperature (20 °C) to observe the temperature-sensitive CLC microspheres. From 60 to 20 °C, the CLC microspheres in the PVA/CLC films are in the cholesteric liquid crystalline phase, leading to a stable bright green appearance across the temperature range (Figure 3c–g). When the temperature reaches 70 °C, the green color fades as the liquid crystalline structure breaks down and shifts to an isotropic phase (Figure 3h). It is noteworthy that the transition temperature to the isotropic phase is higher for the CLC microspheres encapsulated in the PVA/CLC films (70 °C) than for the pure CLC films (60 °C). The result of more stabilized CLC microspheres in the PVA films can be attributed to the protection by polymer chains of PVA. The increased transition temperature could be attributed to the confinement effects from the PVA encapsulation. During the phase transition from the cholesteric liquid crystalline state to the isotropic state, the molecular kinetic energy of the CLC increases, leading to a widening of the intermolecular distances and thermal expansion. The confinement from PVA encapsulation, however, restricts this expansion, thereby stabilizing the cholesteric liquid crystalline phase over an extended temperature range. The increased transition temperature is consistent with the observed increase in the reflection wavelength.

The design principle of polymer films with liquid crystals involves using polymers that can be stabilized in a film state with high transparency along with solvents that can dissolve the polymers but not the liquid crystals. In addition to PVA, polystyrene (PS) and commercial cholesteryl oleyl carbonate (COC) are also used to prepare PS/COC films for evaluating whether other polymers can fulfill the same function as PVA to prevent liquid crystal leakage. PS/COC films are prepared by spin-coating a 15 wt % PS/COC solution. After the PS films are selectively removed by cyclohexane, COC microspheres are founded to be intact, as shown in the SEM images (Figure S2). The results suggest that PS, which is more hydrophobic than PVA, can also fulfill a role similar to that of PVA in preventing liquid crystal leakage and in stabilizing liquid crystal microspheres.

**Figure 4:**
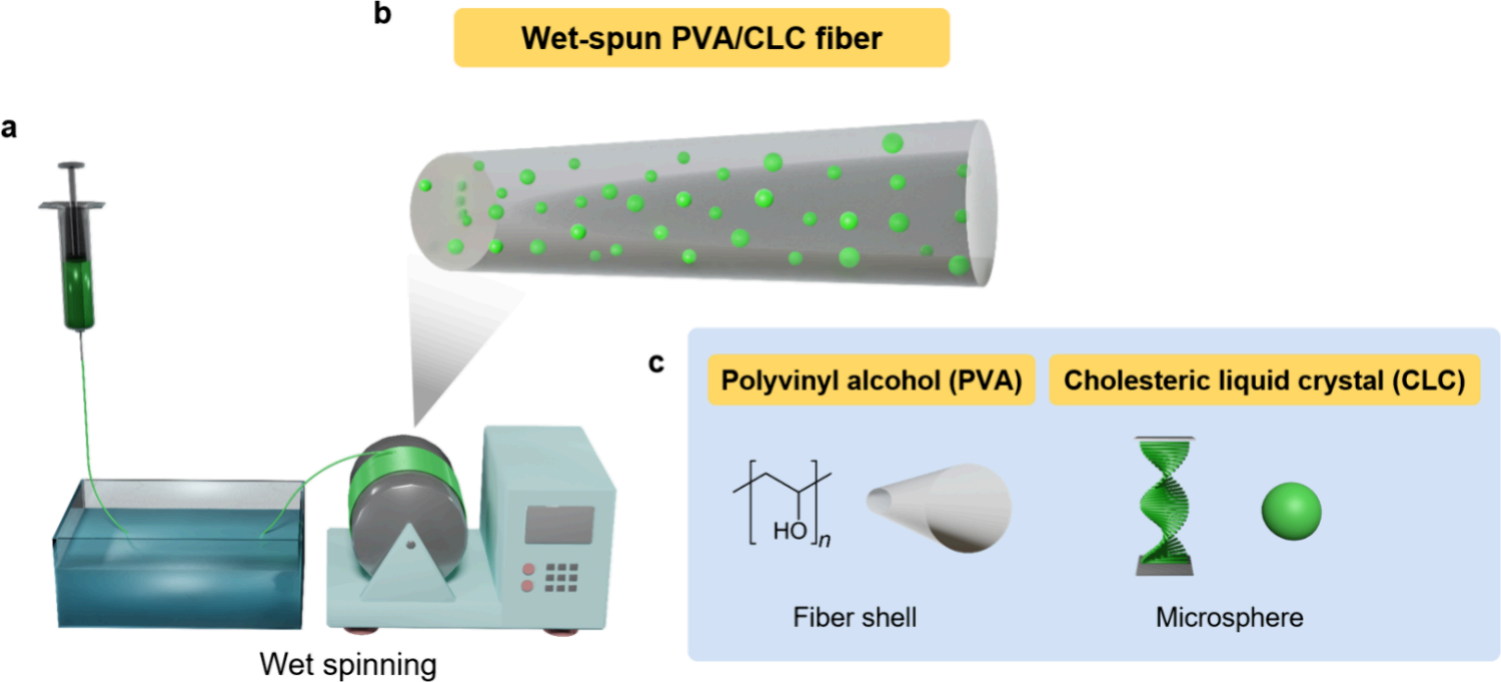
Schematic illustration of the wet-spinning technique (a), PVA/CLC fibers (b), and the constituent materials PVA and CLC (c).

After exploring the behavior of CLC within the PVA matrix in the form of films, we proceed to fabricate fibers using the wet-spinning technique (Figure 4). Initially, the PVA/CLC solution is prepared as per the established procedure, but with a higher PVA concentration of 22 wt %. A higher concentration of PVA solution is utilized to prepare the PVA/CLC fibers because as the PVA concentrations increase, the PVA morphologies become more well-sustained, as illustrated in Figure S3. The prepared PVA/CLC blend is then extruded through a stainless steel needle, and the solution is deposited into the acetone-containing coagulation bath. The acetone bath promotes the coagulation of the fibers, facilitated by the poor solubility of PVA in acetone and the encapsulation of the CLC. The emerging fibers are collected on a rotating drum. After collection, the fibers are left to dry overnight under ambient conditions.

**Figure 5:**
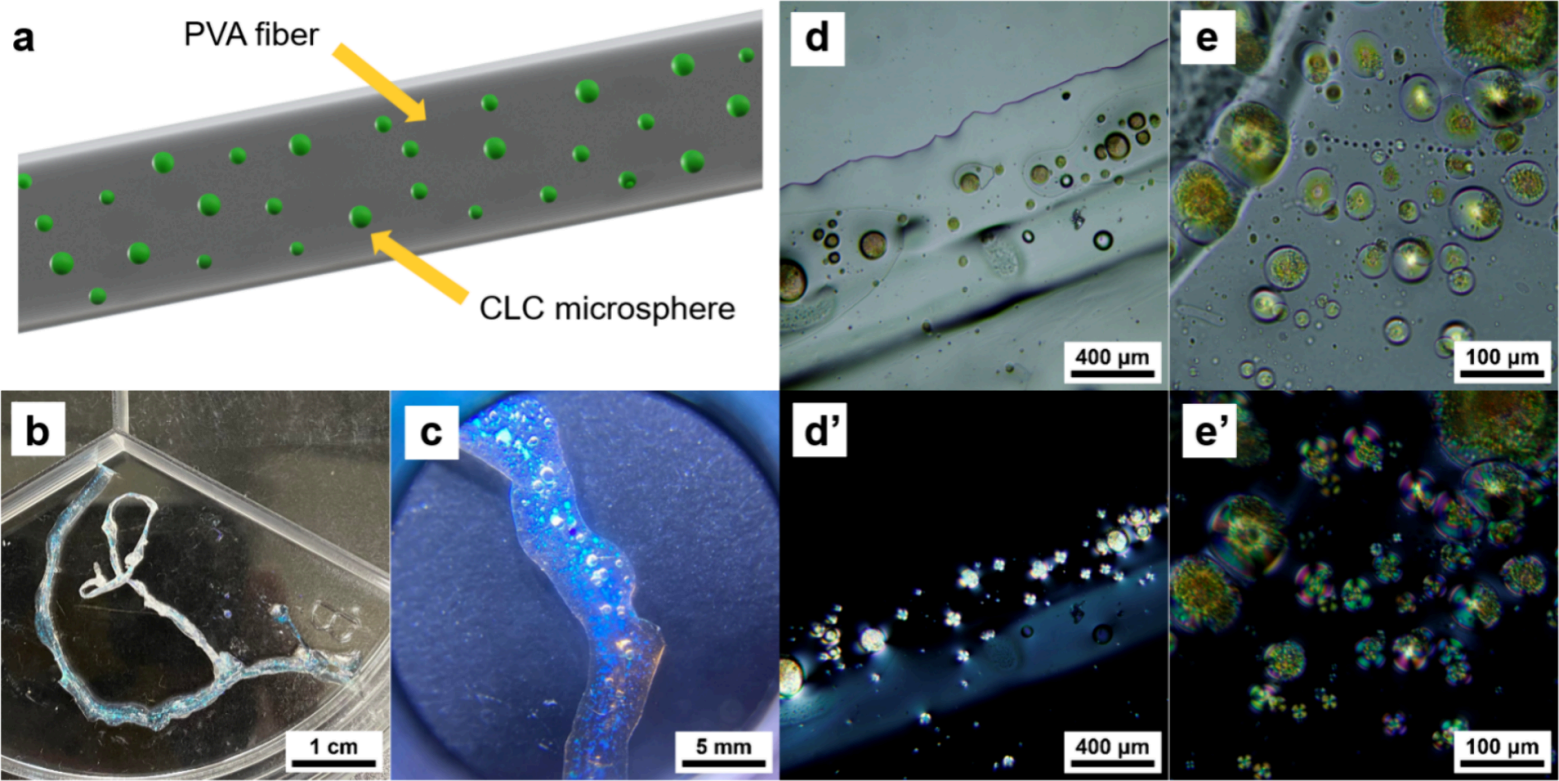
(a) Schematic illustration of wet-spun PVA/CLC fibers. Optical images of the PVA/CLC fiber under normal lighting (b) and under UV light illumination (c). Transmission optical microscopy images (d, e) and polarization optical microscopy images (d′, e′) of the PVA/CLC fibers.

Using the wet-spinning technique, we successfully fabricated PVA/CLC fibers (Figure 5a,b). The CLC microspheres are encapsulated inside the PVA fibers because of the phase separation. Under the white light irradiation, a bright green reflective color is observed. The encapsulated CLC microspheres, however, can also be seen under illumination of UV light (Figure 5c), indicating that the CLC microspheres might be used in the field of light-responsive materials. It should be noted that the absence of these microspheres on the fiber surfaces can be linked to the use of acetone as the coagulating solvent for PVA during the wet-spinning process. The acetone effectively washes away exposed CLC and solidifies the PVA, reducing the chance of CLC leakage in the PVA. Although acetone is a good solvent for CLC, the encapsulated CLC microspheres remain protected because of PVA encapsulation. Under the transmission optical microscope (Figure 5d,e), CLC microspheres are seen dispersed within the PVA matrix, a behavior consistent with what is observed in the PVA/CLC films. Additional polarization microscopy images in Figures 5d′ and 5e′ reveal the Maltese cross patterns, which are characteristics of birefringent materials. In the previous study, Belmonte et al. have demonstrated an effective approach to fabricating angular-independent reflective coatings using photonic cholesteric liquid crystal particles, which show the Maltese cross patterns.^20^ Therefore, the result in this work confirms that the CLC microspheres are still in the cholesteric liquid crystal phase. Furthermore, as the sizes of the liquid crystal microspheres increase, the interference patterns within the Maltese cross become more complex and less distinct.

**Figure 6:**
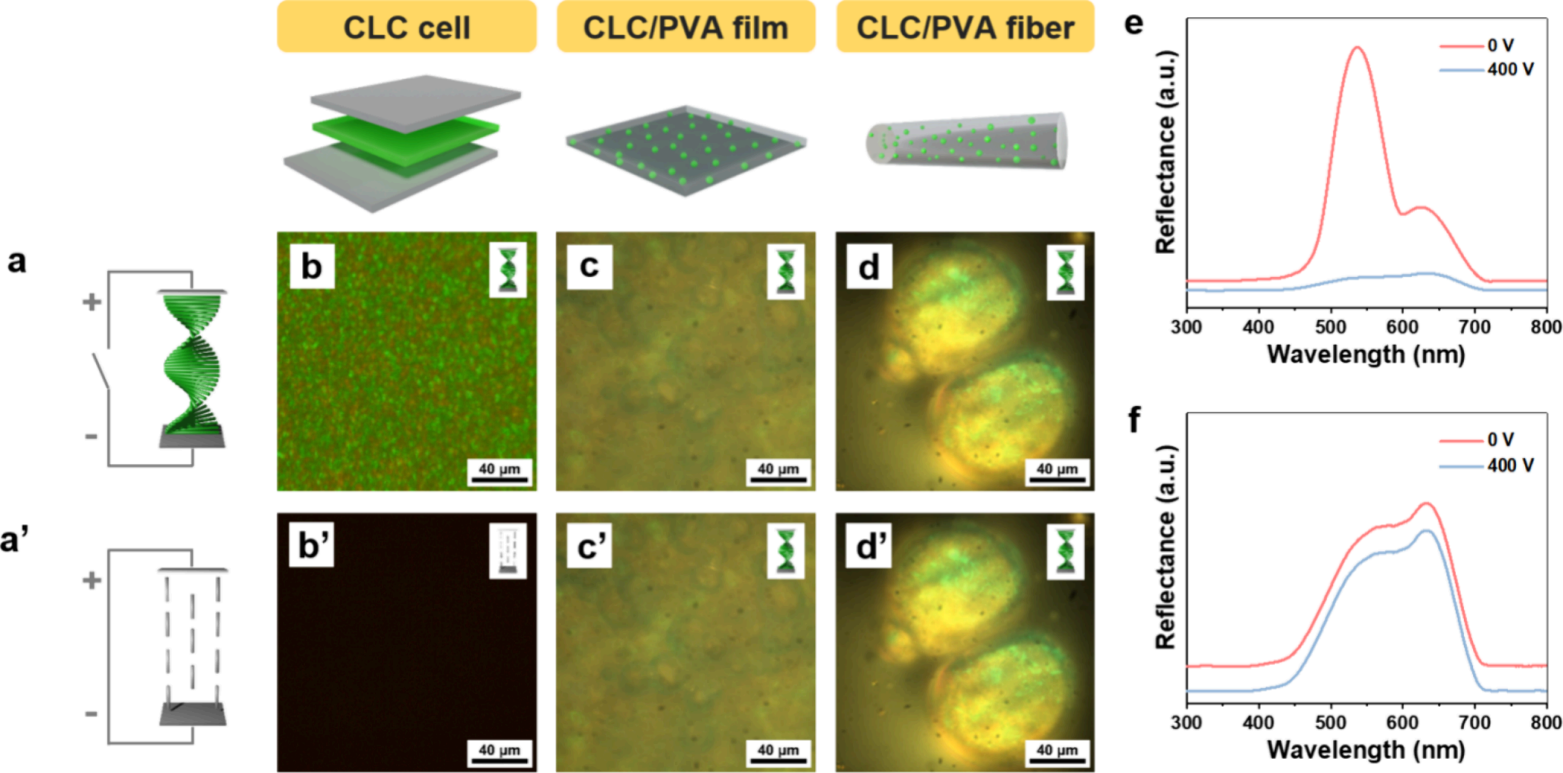
Schematic representations of the cholesteric liquid crystal structures under 0 (a) and 400 V (a′). Reflective optical images of the CLC cell (b), PVA/CLC film (c), and PVA/CLC fiber (d) with no voltage applied. Reflective optical images of the CLC cell (b′), PVA/CLC film (c′), and PVA/CLC fiber (d′) with 400 V applied. Reflectance spectra of the CLC cell (e) and PVA/CLC film (f) under 0 and 400 V.

To investigate the optical property changes of the CLC under the influence of an electric field, a voltage of 400 V (10.8 V/μm) perpendicular to the normality of the CLC film is applied. When no voltage is applied, the CLC is in a cholesteric liquid crystalline state, resulting in a bright green appearance (Figure 6b). With the applied voltage, the liquid crystal molecules align parallel to the electric field, allowing visible light to pass through without noticeable reflection, rendering the CLC film to appear black under an optical microscope (Figure 6b′). As shown in Figure 6e, a low reflection in the visible spectrum is observed for the CLC film under this electric field. PVA/CLC films are sandwiched between 2 conductive ITO glasses for the observation of the electric field effects. The distance between 2 glasses is 500 μm. No color change nor molecular reorientation is observed when the voltage of 400 V is applied (Figure 6c,c′,e). The same holds true when the voltage is increased to 1000 V. The voltage cannot be further increased, owing to the risk of arcing and combustion. Given that an electric field of 10.8 V/μm is required to alter the state of CLC, the voltage needed for 500 μm of a PVA/CLC film should be 5400 V. Similarly, no color change is observed when 400 V is applied to the PVA/CLC wet-spun fibers (Figure 6d,d′).

## Conclusions

In conclusion, we investigate the feasibility of incorporating CLC microspheres into polymer matrix using the nonsolvent and phase separation method. The combination of the polar solvents PVA and CLC results in the formation of CLC microspheres in the PVA matrix. The PVA/CLC films are characterized through optical and polarized microscopy. Thermal testing indicates that the CLC microspheres demonstrate superior thermal stability when encapsulated in the PVA matrix compared with the CLC films. Furthermore, the wet-spinning technique is implemented to produce the PVA/CLC fibers. Electrical tests reveal the good color stability of both the PVA/CLC films and fibers. The effectiveness of the nonsolvent and phase separation method has been demonstrated in producing thermally and electrically stable liquid crystal polymer films and fibers, opening doors for applications in smart textiles, display technologies, and medical devices.

## Supporting Information Available

Real images of NYCL-15% CLC microspheres in H_2_O/DMSO with various non-cosolvent compositions; SEM images of PS/COC films after spin-coating 15 wt % PS/COC in DMF, followed by removing PS with cyclohexane at different times; real images and optical images of the PVA/CLC fibers with different concentrations of PVA (PDF)
