# Supplementary material for: Fabrication of Polymer/Cholesteric Liquid Crystal Films and Fibers Using the Nonsolvent and Phase Separation Method
Source: Langmuir. 2024 Jun 25;40(27):14166–72. doi: 10.1021/acs.langmuir.4c01759 (PMC11238578; doi:10.1021/acs.langmuir.4c01759)
Supplement: Supplementary file 1 — la4c01759_si_001.pdf [file la4c01759_si_001.pdf]

Supporting Information

Fabrication of Polymer/Cholesteric Liquid Crystal Films and Fibers Using the Nonsolvent and Phase Separation Method

Tzu-Hsun Kao,[1] Hsun-Hao Hsu,[1] Jui-Juin Chen,[2] Lin-Ruei Lee,[1] and Hui-Yu Chen,[2]* Jiun-Tai Chen[1,3]*

[1]Department of Applied Chemistry, National Yang Ming Chiao Tung University, Hsinchu 300093, Taiwan

[2]Department of Physics, National Chung Hsing University, Taichung City 402204, Taiwan

[3]Center for Emergent Functional Matter Science, National Yang Ming Chiao Tung University, Hsinchu 300093, Taiwan

*To whom correspondences should be addressed. Email: huiyuchen@nchu.edu.tw (H.-Y. Chen) and jtchen@nycu.edu.tw (J.-T. Chen).

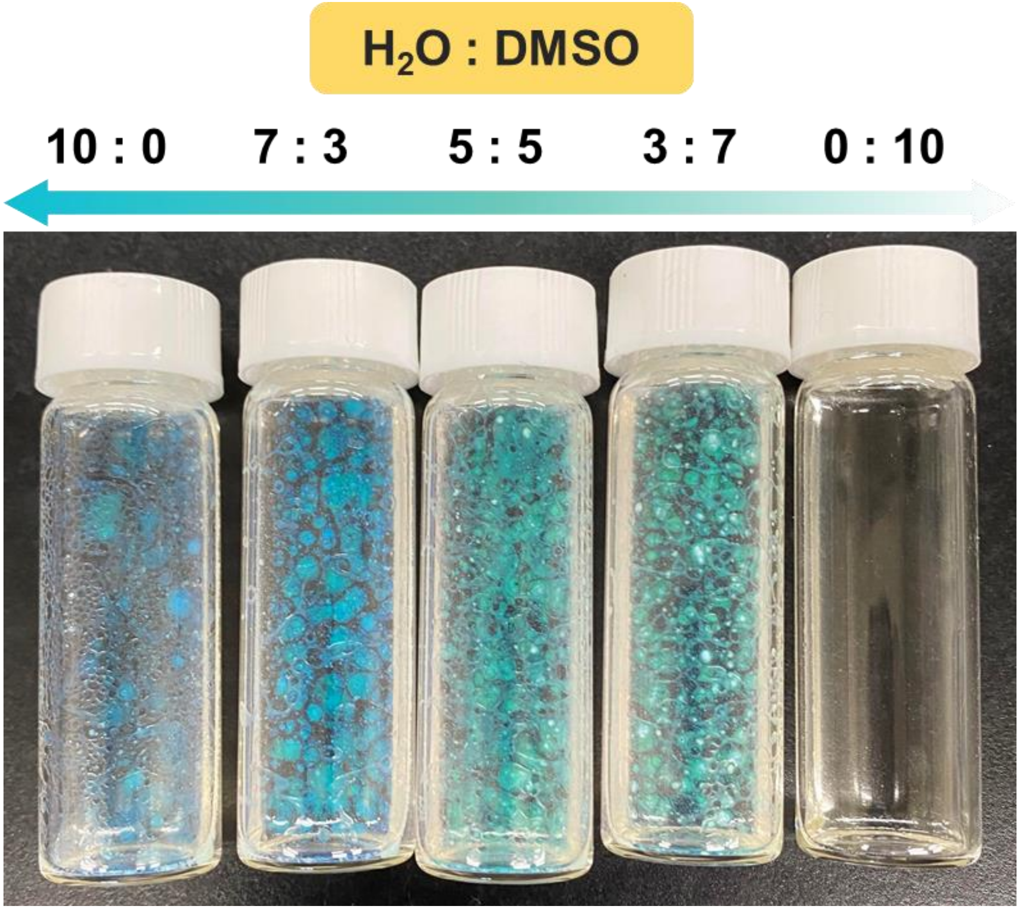

Figure S1. Real image of NYCL-15% CLC microspheres in the H_2O/DMSO cononsolvents with different non-solvent compositions.

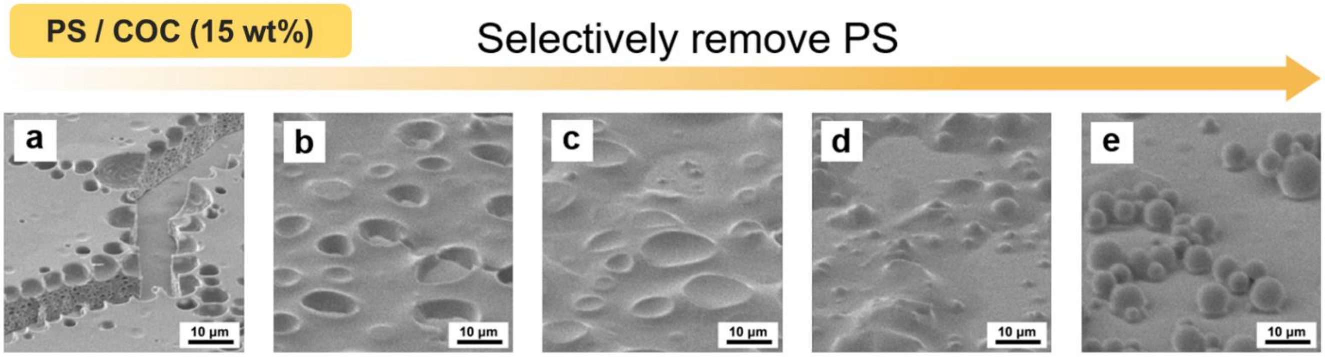

Figure S2. (a-e) SEM images of PS/COC films made by spin-coating the solution of 15 wt % PS/COC in DMF, followed by gradually removing the PS films using cyclohexane with different times.

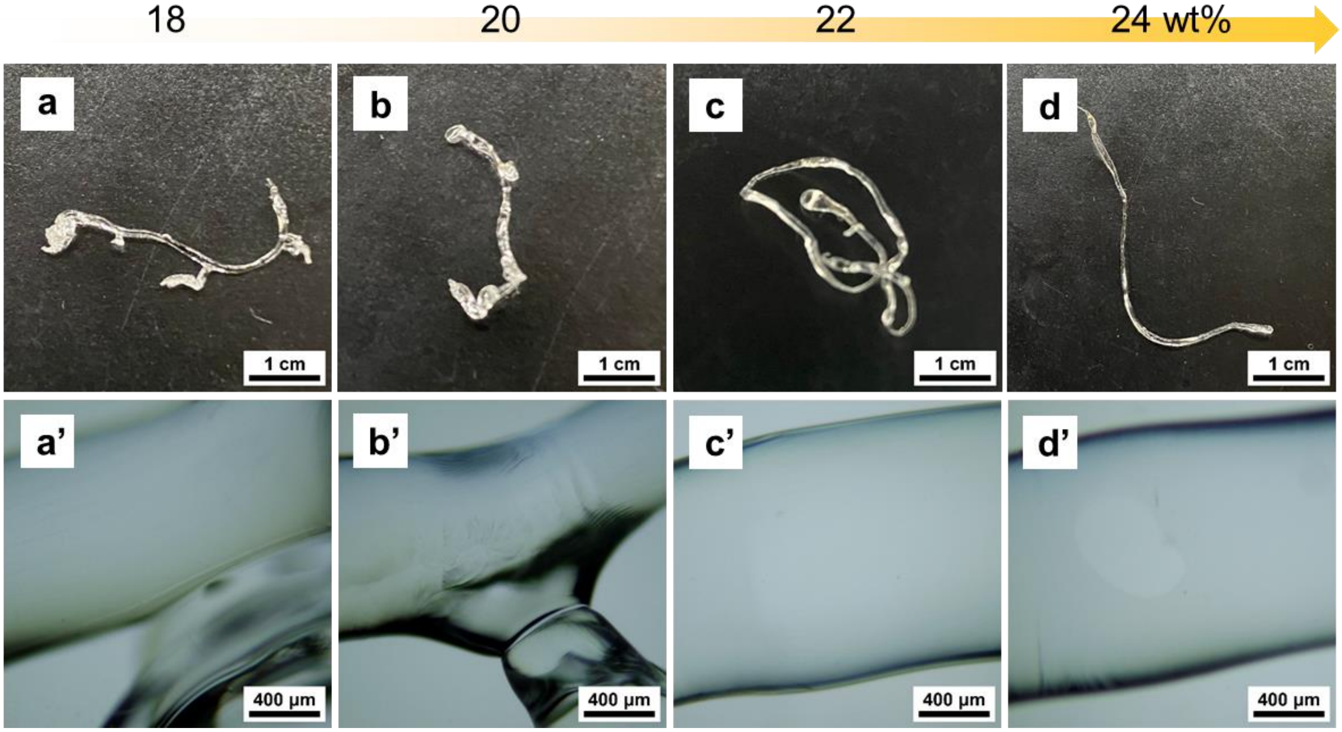

Figure S3. (a-d) Real images of the PVA/CLC fibers: (a) 18, (b) 20, (c) 22, and (d) 24 wt % of PVA. (a'-d') Optical images of the PVA/CLC fibers: (a) 18, (b) 20, (c) 22, and (d) 24 wt % of PVA.
